# Facile Synthesis of Fluorescent Carbon Quantum Dots with High Product Yield Using a Solid-Phase Strategy

**DOI:** 10.3390/molecules29225317

**Published:** 2024-11-12

**Authors:** Haitao Ren, Fan Qi, Xiangbo Feng, Jiaxiang Liu, Yuzhen Zhao

**Affiliations:** 1 Technological Institute of Materials & Energy Science (TIMES), Xijing University, Xi’an 710123, China; 2 State Key Laboratory of Medicinal Chemical Biology, College of Pharmacy, Nankai University, Tianjin 300071, China

**Keywords:** solid-phase synthesis, carbon quantum dots, high product yield, fluorescence

## Abstract

The liquid-phase method is the most commonly utilized strategy for synthesizing fluorescent carbon quantum dots (CQDs). However, the liquid-phase synthesis of CQDs faces challenges such as low yield, complex purification, and the use of toxic solvents, which limit large-scale production and practical applications. In this study, fluorescent CQDs with a high product yield of 78% were synthesized using glucose as a carbon source through a green and facile one-step solid-phase approach, without solvents or post-treatment. A systematic study of the structure and fluorescence properties of the synthesized CQDs was conducted using various characterization techniques. The results indicated that the mean size of obtained CQDs was 4.1 nm, and that their surface had abundant oxygen-containing functional groups, resulting in favorable water solubility. The synthesized CQDs exhibited excitation-dependent fluorescence, with optimal excitation and emission wavelengths at 358 and 455 nm, respectively. Additionally, the CQDs solution showed bright blue fluorescence under 365 nm UV light, with a quantum yield of 6.21% and a fluorescence lifetime of 3.02 ns. This study offers valuable insights into the green and efficient synthesis of fluorescent CQDs powder.

## 1. Introduction

Quantum dots are semiconductor nanocrystal materials that exhibit tunable optical and electronic properties due to quantum confinement effects [1]. Carbon quantum dots (CQDs), a type of quantum dots, were discovered by scientists in 2004 while purifying and isolating single-walled carbon nanotubes [2]. Later, in 2006, scientists named these fluorescent carbon nanoparticles “carbon quantum dots” [3]. Typically, intrinsic CQDs are composed of carbon and oxygen elements, with typical particle sizes of less than 10 nm [4]. Their structure consists of sp2 and sp3 hybridized carbon nuclei and organic functional groups on the surface of the carbon nuclei [5]. Compared to the widely reported inorganic quantum dots (e.g., CdS, CdSe, SnS, and CdTe) that exhibit low hydrophilicity, challenging production, inadequate stability, and elevated toxicity [6,7], CQDs demonstrate unique advantages (i.e., low preparation cost, small size, excellent fluorescence performance, good hydrophilicity, high chemical stability, ultra-low toxicity, and favorable electronic conductivity) [8,9,10,11,12], suggesting that CQDs are a favorable substitute for semiconductor quantum dots based on toxic metals. At present, CQDs have been widely used in several fields including biological imaging, anti-counterfeiting printing, optoelectronic devices, photocatalysis, fluorescence sensing, plant growth, etc. [13,14].

Top-down and bottom-up methodologies are commonly employed in the synthesis of CQDs, with the choice depending on the precursor types and preparation procedures [15]. Early in the study of CQDs, techniques like arc discharge, laser ablation, electrochemical stripping, and chemical oxidation were employed to break down carbon materials like graphene, coal ash, and fullerene into CQDs [16]. The obtained CQDs usually have low quantum yields and poor water solubility, requiring modification to improve their optical properties, eventually leading to complex post-processing and environmental pollution. Currently, this method is rarely employed to synthesize CQDs. The bottom-up approach is widely utilized for CQDs synthesis, including methods such as hydrothermal, solvothermal, and microwave techniques. Researchers have investigated the polymerizable functional groups of precursor molecules to construct larger carbon-based structures, focusing on processes such as precursor polymerization, crosslinking, and carbonization [17,18]. Although these strategies have shown promise in high-quality fluorescent CQDs synthesis, there are still several issues with these methods: (1) They require the usage of multiple solvents, resulting in complex post-processing steps including centralization, filtration, chromography, dialysis, electrophoresis, etc.; (2) the reaction and post-treatment processes generate a large amount of organic waste liquid, which poses potential environmental risks; (3) more importantly, the product yield of the CQDs synthesized through these methods is relatively low (about 20%), further limiting the large-scale preparation and practical application of CQDs. Therefore, there is an urgent need to develop greener and more efficient processes for synthesizing fluorescent CQDs with high product yield.

**Scheme 1:**
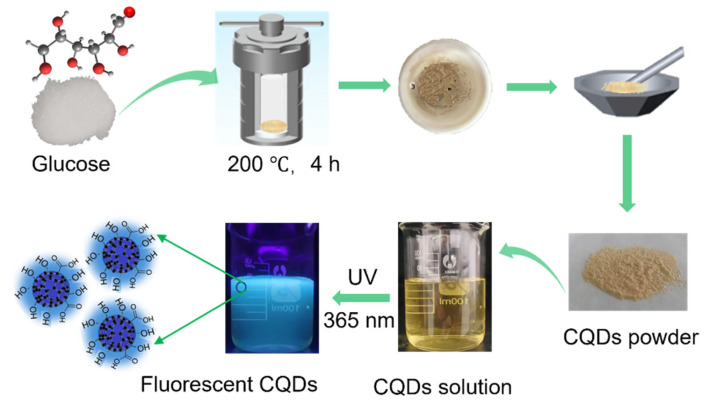
Flow chart of one-step solid-phase synthesis of fluorescent CQDs employing glucose as a carbon source.

Glucose is a green and low-cost precursor for the preparation of CQDs [19,20]. In the present study, we synthesized fluorescent CQDs by a green and efficient one-step solid-phase strategy with glucose as the carbon source, requiring no solvents or post-treatment (Scheme 1). The product yield of the synthesized CQDs was as high as 78%, which is significantly higher than those reported in previous studies. Furthermore, the structure and fluorescence behavior of the synthesized CQDs were systematically studied using various characterization techniques, elucidating the relationship between their structure and fluorescence properties. This work provides insights into the green and convenient synthesis of fluorescent CQDs with high product yield from glucose-based feedstocks, which is significant for promoting the large-scale synthesis and practical applications of CQDs.

## 2. Results and Discussion

### 2.1. Structural Analysis of the Produced CQDs

**Figure 1:**
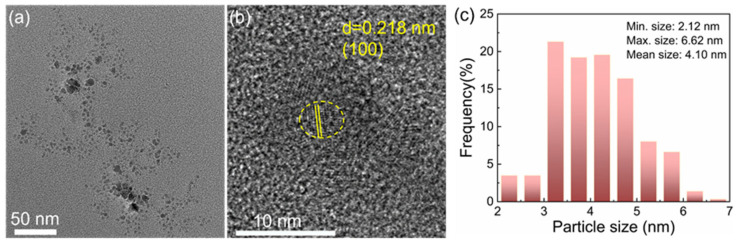
Morphological characterization of CQDs. (**a**) TEM image, (**b**) high-resolution TEM image (Yellow circle area represents the lattice fringe of CQDs), and (**c**) size distribution histogram of the synthesized CQDs.

**Figure 2:**
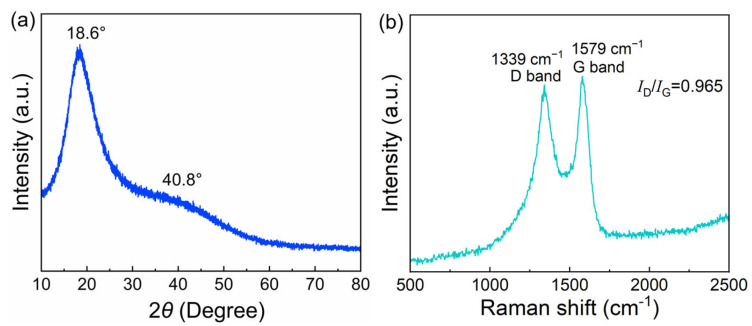
Microstructure of CQDs. (**a**) XRD pattern and (**b**) Raman spectrum of the fabricated CQDs.

The microstructure and particle size of the synthesized CQDs were studied using TEM microscopy. As shown in Figure 1a, the shape of the CQDs is spherical, and the aggregation between particles may be caused by soft aggregation. CQDs crystallinity is an important property, thus the lattice fringes of the prepared CQDs were measured using high-resolution TEM. The associated high-resolution TEM image of the produced CQDs is shown in Figure 1b. These particles have fringe spacings of 0.218 nm (see the yellow circle in Figure 1b), which fit the (100) lattice distance of graphite carbon [21]. The particle size distribution of CQDs was statistically analyzed using Nano Measure 1.2 software. Figure 1c reveals that the size of the CQDs particles ranges from 2.12 to 6.62 nm, with a mean particle size of 4.1 nm. More importantly, the particle size of the CQDs is less than 10 nm, indicating that the synthesized sample is within the size range of quantum dots. In addition, the XRD spectrum of the CQDs (Figure 2a) depicts two broad diffraction peaks near 18.6° and 40.8°, which are consistent with the diffraction peaks of the (002) and (100) crystal planes of carbonaceous nanomaterials with some defects [22]. The Raman spectrum of powdered CQDs was tested to obtain stronger evidence of the intrinsic state of the CQDs carbon nuclei. As depicted in Figure 2b, the Raman spectrum has two characteristic absorption bands at 1339 and 1579 cm^−1^ that correspond to the *D* band (produced from disordered carbon) and the *G* band (derived from graphite carbon), respectively [23]. The intensity ratio of *I*_D_ to *I*_G_ in the CQDs Raman spectrum is 0.965, suggesting a high degree of graphitization and the presence of specific flaws.

**Figure 3:**
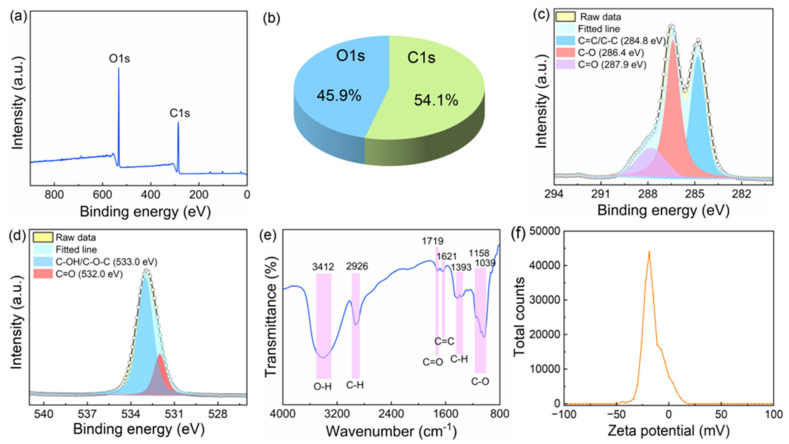
Surface structure of CQDs. (**a**) XPS survey spectrum, (**b**) XPS element content pie chart, (**c**) XPS C1s spectrum, (**d**) XPS O1s spectrum, (**e**) FT-IR spectrum, and (**f**) Zeta potential of CQDs.

The chemical composition and surface state of the prepared CQDs were analyzed utilizing XPS spectroscopy. The XPS full spectrum of the fabricated CQDs is shown in Figure 3a, with two characteristic peaks at 284.8 and 531.1 eV corresponding to the binding energies of C1s and O1s orbitals, respectively. The mass percentages of C and O atoms obtained by XPS were 54.1% and 45.9%, respectively (Figure 3b). The XPS fine spectrum of C1s in CQDs exhibits three different peaks at 284.8, 286.4, and 287.9 eV (Figure 3c), which are attributed to the C=C/C-C, C-O, and C=O bonds, respectively [24]. The XPS fine spectrum of O1s in CQDs reveals two distinct peaks at 532.0 and 533.0 eV (Figure 3d), corresponding to the C=O and C-OH/C-O-C bonds, respectively [25]. In addition, functional groups on the surface of CQDs were identified employing FT-IR spectroscopy.

As shown in Figure 3e, the FT-IR spectra of the CQDs powder exhibit stretching vibration modes of O-H and C-H bonds at 3412 and 2926 cm^−1^, respectively. The distinct absorption bands at 1719 and 1621 cm^−1^ correspond to the vibrations of C=O and C=C bonds, respectively. The absorption band at 1393 cm^−1^ corresponds to the bending vibration mode of the -CH_3_ group [26]. Additionally, the spectral bands at 1039 and 1158 cm^−1^ represent the stretching vibrations of C-O bonds [27]. These results suggest that O-containing groups are effectively anchored on the surface of CQDs. The FT-IR and XPS findings confirm the presence of functional groups such as -COOH and -OH on the surface of the CQDs, which enhance the stability and dispersibility of CQDs in aqueous systems, thus facilitating their practical applications. To gain a deeper understanding of the composition and functional groups on the surface of the CQDs, Zeta potential analysis was performed on the aqueous solution of the synthesized CQDs. As depicted in Figure 3f, the Zeta potential of the CQDs solution (pH 7) was detected to be −15.3 mV, indicating strong electron-rich properties. This is primarily attributed to the abundance of negatively charged groups, such as -OH and -COOH, on the surface of the CQDs. The previous system analysis showed that CQDs with carbon nuclei obtained from glucose using a one-step solid-phase method have certain defects and rich functional groups on their surface. Table 1 compares the characteristics of CQDs synthesized by various methods reported in the literature with those synthesized in this study. It can be seen that the product yield of the CQDs synthesized in this study (78.0%) is significantly higher than that of the reported CQDs in previous literature [28], indicating that the synthesis method developed in this study has great potential for large-scale and efficient synthesis of CQDs. Furthermore, the CQDs synthesized in this study are comparable in properties to CQDs derived from glucose in previous studies (Table 2). Importantly, the CQDs synthesized in this work have a high product yield.

### 2.2. Optical Properties of the Obtained CQDs

**Figure 4:**
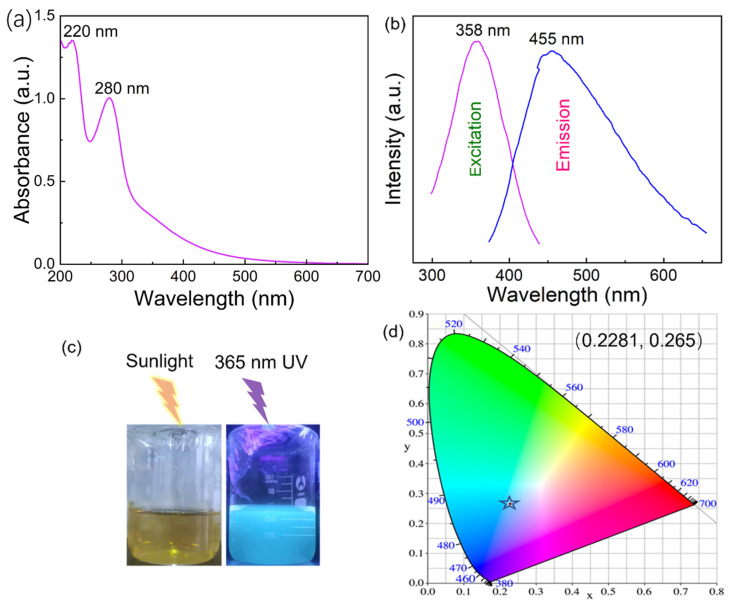
Light absorption behaviors of CQDs. (**a**) UV–vis spectrum, (**b**) excitation and emission spectra of CQDs in water, (**c**) photographs of CQDs suspension under sunlight and at 365 nm under UV lamp, and (**d**) CIE coordinates of the CQDs.

The fluorescence properties of the synthesized CQDs were studied using fluorescence spectroscopy and UV–vis absorption spectroscopy. Dissolving the prepared CQDs powder in water can form a transparent solution, indicating that the synthesized CQDs material is hydrophilic. As displayed in Figure 4a, the UV–vis absorption spectrum exhibits two different absorption peaks at 220 and 280 nm, attributed to the π-π* transition of conjugated C=C and the n-π* transition of aromatic C=O, respectively [43]. Similar to carbon nanoparticles and carbon nanoparticle-based QDs reported in the literature, the CQDs exhibit strong absorption in the UV region, with most of their visible light absorption concentrated in the blue range [44]. The CQDs have broad absorption in the UV–visible region, which is crucial for their application. Figure 4b illustrates that the optimal excitation wavelength for CQDs solution is 358 nm, and the corresponding optimal emission wavelength is 455 nm, suggesting that the CQDs solution emits blue fluorescence. To further identify the fluorescence color of the CQDs solution under UV excitation, the synthesized CQDs powder was dissolved in water to form a transparent liquid. As revealed in Figure 4c, the CQDs solution exhibits brilliant blue fluorescence at 365 nm under a UV lamp, but appears brownish yellow in natural light. Further verification of the fluorescence color emitted by the CQDs solution was conducted through color coordinates. The optimal emission spectrum data of the CQDs solution was imported into the CIE1931 color coordinate software. As displayed in Figure 4d, the resulting color coordinates of the CQDs solution are (0.2281, 0.265), indicating typical blue fluorescence.

**Figure 5:**
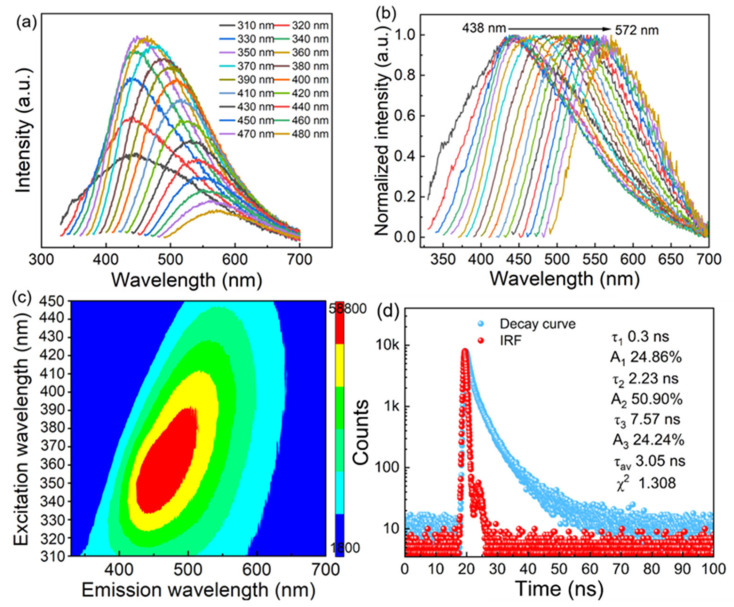
Fluorescence properties of CQDs. (**a**) Excitation-dependent fluorescence spectra, (**b**) corresponding 3D fluorescence spectra (the excitation wavelengths range from 310 to 480 nm with an interval of 10 nm), (**c**) normalized fluorescence spectra, and (**d**) fluorescence lifetime curve of CQDs. Red line denotes our system’s instrument response function (IRF).

Figure 5a displays the fluorescence spectra of the CQDs solution at various excitation wavelengths (310–480 nm). The CQDs exhibit a progressive increase in fluorescence intensity as the excitation wavelength increases from 310 to 350 nm. However, as the excitation wavelength further increases from 350 to 480 nm, the CQDs fluorescence intensity gradually drops. Obviously, the fluorescence emission of CQDs is heavily dependent on the excitation wavelength, and this excitation-dependent fluorescence phenomenon is similar to most previously reported results [45]. The normalized fluorescence spectra further confirmed the excitation-dependent fluorescence properties of the CQDs. Figure 5b shows that when the excitation wavelength of the CQDs increases from 310 to 480 nm, the center of the corresponding emission spectrum increases from 438 to 572 nm. The tunable fluorescence properties of CQDs may be attributed to the non-uniformity of particle size and the large number of surface emission states formed by functional hydroxyl and carboxyl groups [46,47]. The optical properties of the CQDs were determined by the carbon-core structure and its surface functional groups, providing an effective solution for regulating the fluorescence properties of CQDs. In addition, the 3D fluorescence contour map of the CQDs showed many contours (corresponding to the core and surface) appearing at the same emission center of 455 nm, further demonstrating the excellent fluorescence emission performance of the synthesized CQDs (Figure 5c). Owing to its excitation-dependent properties, the fluorescence lifetime of the CQDs was also evaluated. As exhibited by Figure 5d, the fluorescence lifetime of the CQDs solution was found to be 3.05 ns, based on three exponential fittings. This nanosecond-level lifetime indicates that the synthesized CQDs have favorable fluorescence properties [48]. In particular, the τ_1_ of the CQDs is 0.3 ns, with a percentage of 24.86%; the τ_2_ of the CQDs is 2.23 ns, with a percentage of 50.9%; and the τ_3_ of the CQDs is 7.57 ns, with a percentage of 24.24%. The short-lived τ_1_ can be related to the intrinsic state transitions of the carbon-core states of the CQDs, while long-lived τ_2_ and τ_3_ may be related to radiative or non-radiative recombination from surface states of the CQDs at the same emission wavelength [48,49]. In addition, the quantum yield of the CQDs solution was measured using the absolute method as reported in previous literature. As shown in Figure S1, the absolute fluorescence quantum yield of the CQDs solution was calculated to be approximately 6.21%, which is comparable to that of unmodified CQDs reported in the literature [50,51].

**Figure 6:**
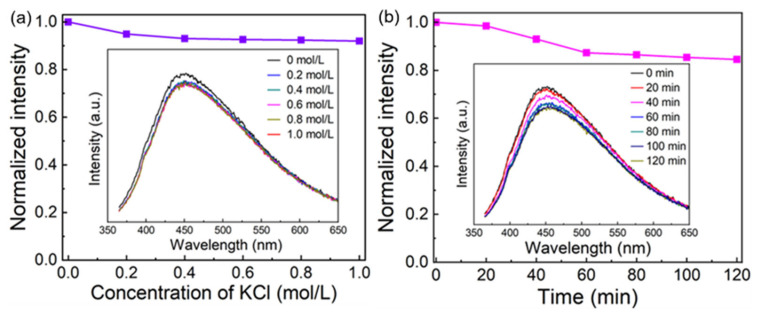
The influence of different factors on the fluorescence of CQDs. (**a**) The impact of varying KCl concentrations and (**b**) UV lamp irradiation period on CQDs fluorescence intensity (λex = 358 nm).

Furthermore, the effects of varying salt ion concentrations and UV irradiation durations on the fluorescence stability of the synthesized CQDs were investigated. As shown in Figure 6a, the CQDs exhibited strong resistance to salt ions, with the fluorescence intensity of the CQDs solution remaining nearly unchanged even when the KCl concentration reaches 1.0 mol/L. Figure 6b shows that the CQDs also demonstrated significant resistance to photobleaching, as the fluorescence intensity of the CQDs solution remains stable after 120 min of continuous irradiation at 365 nm under a UV lamp. These characteristics indicate that the obtained CQDs have stable fluorescence properties under complex environmental conditions, which is of great significance for the practical applications of CQDs. Given the small size, high product yield, excellent fluorescence performance, rich surface functional groups, and environmentally friendly characteristics of CQDs, the synthesis method developed in this study provides a meaningful reference for green, low-cost, and large-scale production of fluorescent CQDs.

## 3. Materials and Methods

### 3.1. Materials

D-anhydrous glucose (C_6_H_12_O_6_, AR) was supplied from Shanghai McLean Biochemical Technology Co., Ltd. (Shanghai, China). Potassium chloride (KCl, anhydrous grade, ≥99%) and potassium bromide (KBr, ≥99.0%) were purchased from Shanghai Aladdin Biochemical Technology Co., Ltd. (Shanghai, China). Ultrapure water (18 m Ω/cm, 25 °C) was utilized as a solvent throughout the entire experimental process.

### 3.2. Production of CQDs

High-yield CQDs powder was synthesized using a one-step solid-phase method. Firstly, 2.0 mmol of glucose powder was spread evenly into a 100 mL PTFE liner. Then, the liner was placed in an electric hot-air drying oven for a solid-phase reaction at 200 °C for 4 h. After the reaction was completed, the resulting brownish-yellow powder was ground in an agate mortar for 30 min to produce a fine CQDs powder. The CQDs powder was then added to ultrapure water and sonicated for 1 h to yield a transparent yellow-brown CQDs solution. The final product yield of the CQDs powder was calculated using Equation (1):

(1) $$Y=\frac{W_{t}}{W_{0}}$$

where *Y* presents the product yield of CQDs, *W*_0_ denotes the mass of glucose, and *W*_t_ refers to the mass of CQDs powder. To reduce errors, the experiment was repeated three times and the average product yield of CQDs was calculated to be 78.0%.

### 3.3. Characterizations

The morphology of CQDs was analyzed by way of a JEM-F200 transmission electron microscope (TEM) manufactured by JEOL in Tokyo, Japan, with an acceleration voltage of 200 kV and sample preparation using ultra-thin carbon film. The elemental composition and surface chemical states of CQDs were determined utilizing a Thermo ESCALAB 250XI X-ray photoelectron spectroscopy (XPS) produced by Thermo Fisher Scientific in Waltham, MA, USA. The excitation source was 250 W Al target Ka rays, and the binding energy of the elements was corrected based on C1s (284.8 eV). The Zeta potential of CQDs solution was tested using the Malvern Zetasizer Nano ZS90 Zeta potential meter manufactured by the British company Malvern (Malvern, UK). The surface functional groups of the CQDs powder were tested using a Bruker (Verte70 V) Fourier (Bruker, Billerica, MA, USA) transform infrared spectrometer (FT-IR) from Germany, and measured by pressing in KBr medium within the range of 800–4000 cm^−1^. The UV–vis absorption spectra of CQDs solution were measured employing a UV-2600 UV–vis spectrophotometer manufactured by Shimadzu Corporation in Kyoto, Japan. The crystal structure of the CQDs powder was tested utilizing a Smart Lab 9 kW X-ray diffractometer (XRD) manufactured by Rigaku Corporation in Tokyo, Japan. The structure of the powder CQDs’ carbon nuclei was analyzed using a HORIBA LabRAM (Horiba, Kyoto, Japan) micro-confocal Raman spectrometer, equipped with a 532 nm laser wavelength.

### 3.4. Fluorescence Testing of CQDs

The fluorescence attenuation curve of CQDs aqueous solution was tested on an Edinburgh Instruments FS5 fluorescence spectrometer with a laser wavelength of 340 nm. The fluorescence attenuation curve was fitted using the following three-index model Equation (2) [52]:

(2) $$R\left(t\right)=\alpha_{1}\exp\left(\frac{-t}{\tau_{1}}\right)+\alpha_{2}\exp\left(\frac{-t}{\tau_{2}}\right)+\alpha_{3}\exp\left(\frac{-t}{\tau_{3}}\right)$$

where the fractional contributions of the three transition channels corresponding to decay lifetimes τ_1_, τ_2_, and τ_3_ to the fluorescence emission of CQDs are represented by α_1_, α_2_, and α_3_.

The average fluorescence lifetime (τ_av_) of CQDs was calculated using Equation (3):

(3) $$\tau_{\mathrm{av}}=\tau_{1}A_{1}+\tau_{2}A_{2}+\tau_{3}A_{3\;}$$

where A_1_, A_2_, and A_3_ represent amplitude fractions, while τ_1_, τ_2_, and τ_3_ denote the corresponding lifetimes of each component.

In addition, the excitation and emission spectra of the CQDs solution were measured using the same Edinburgh Instruments FS5 fluorescence spectrometer (Edinburgh Instruments Ltd., Livingston, UK), with excitation wavelengths ranging from 310 to 480 nm. To investigate the effect of different salt ion concentrations on the fluorescence intensity of the CQDs solution, KCl solid powders of different masses were added to the obtained 3 mL CQDs solution, where the concentration of KCl ranged from 0.0 to 1.0 mol/L. Then, the CQDs solutions were placed under a UV lamp at 365 nm for different durations to study the potential of the CQDs solutions against photobleaching.

## 4. Conclusions

In summary, the synthesis of CQDs via the liquid-phase method faces issues such as a complex post-treatment process and low product yield. This study developed a strategy to synthesize fluorescent CQDs from glucose using a solid-phase strategy that requires no solvents or post-processing, and that is characterized by simplicity, environmental friendliness, and high efficiency. The product yield of the fluorescent CQDs obtained using this strategy is as high as 78%, which is significantly higher than previously reported results. Analysis shows that the mean particle size of the CQDs is 4.1 nm, with abundant oxygen-containing functional groups (-OH, -COOH) on the surface, endowing them with favorable fluorescence and hydrophilicity. Additionally, the CQDs exhibit excitation-dependent fluorescence, with optimal excitation and emission wavelengths at 358 and 455 nm, respectively. Under 365 nm UV light, the CQDs solution emits bright blue fluorescence with a quantum yield of 6.21%. The prepared CQDs show stable fluorescence under high salt ion concentration (1.0 mol/L) and extended UV irradiation (120 min). This work provides a novel approach for the efficient and environmentally friendly synthesis of fluorescent CQDs with high product yield. The obtained CQDs are expected to be applied in anti-corrosion, photocatalysis, optoelectronic devices, sensing, and other fields. Despite the high product yield of the CQDs, their fluorescence quantum yield is relatively low. Enhancing the fluorescence quantum yield of CQDs through surface modifications in solid-phase synthesis will be a primary focus of future research.

## Figures and Tables

**Table 1:** Comparison of CQDs properties derived from other precursors and methods.

Precursor	Material	Synthetic Method	Wavelength/nm	Production Yield/%	Refs.
Ethanolamine, phosphoric acid	N, P-CQDs	Microwave hydrothermal	340/417	15.00	[29]
Fructose	N, S-CQDs	Micro-plasma treatment	370/450	16.60	[30]
Fresh lemon juice	CQDs	Hydrothermal treatment	410/482	0.10	[31]
Ethylene gas	CQDs	Plasma assisted deposition	360/420	10.00	[32]
C60	CQDs	Thermal decomposition	400/510	15.00	[33]
Egg	N-CQDs	Plasma-induced method	360/420	5.96	[34]
Food waste, ethanol	CQDs	Ultrasound irradiation	330/400	0.12	[35]
Succinic acid, trisamine	N-CQDs	Microwave-assisted synthesis	340/422	17.30	[36]
Lysine	N-CQDs	Microwave pyrolysis	360/432	30.00	[37]
Glucose	CQDs	One-step solid-phase method	358/455	78.0	This work

**Table 2:** Comparison of CQDs properties derived from glucose.

Material	Synthetic Method	Wavelength/nm	Mean Size/nm	Quantum Yield/%	Refs.
GQDs	Pyrolysis at 250 °C for 25 min	350/426	8.5	NA	[38]
CQDs	Ultrasonic treatment at 400 W for 4 h	350/435	3.0	4.26	[39]
CQDs	Hydrothermal treatment at 200 °C for 8 h	360/440	3.5	NA	[40]
CQDs	Microwave method at 800 W for 4 min	355/450	2.4	4.5	[41]
N-CQDs	Microwave method at 700 W for 5 min	370/457	4.0	12.4	[42]
CQDs	Hydrothermal approach at 200 °C for 12 h	370/505	3.8	1.38	[19]
CQDs	Hydrothermal treatment at 220 °C for 10 h	320/420	3.0	NA	[20]
CQDs	One-step solid-phase method	358/455	4.1	6.21	This work

NA: Not Available.

## Data Availability

All data are included in the article.

## Supplementary Materials

The following supporting information can be downloaded at: https://www.mdpi.com/article/10.3390/molecules29225317/s1, Figure S1: Calculation of absolute PL quantum yield of CQDs.
