# Supplementary material for: Facile Synthesis of Fluorescent Carbon Quantum Dots with High Product Yield Using a Solid-Phase Strategy
Source: Molecules. 2024 Nov 12;29(22):5317. doi: 10.3390/molecules29225317 (PMC11596220; doi:10.3390/molecules29225317)
Supplement: Supplementary file 1 [file molecules-29-05317-s001.zip › molecules-3224648-supplementary.pdf]

Supplementary Information

Facile Synthesis of Fluorescent Carbon Quantum Dots with High Product Yield Using a Solid-Phase Strategy

Haitao Ren [1,*], Fan Qi [2], Xiangbo Feng [1], Jiaxiang Liu [1] and Yuzhen Zhao [1,*]

[1] Technological Institute of Materials & Energy Science (TIMES), Xijing University, Xi'an 710123, China
[2] State Key Laboratory of Medicinal Chemical Biology, College of Pharmacy, Nankai University, Tianjin 300071, China
* Correspondence: bs210311007@sust.edu.cn (H.R.); zyz19870226@163.com (Y.Z.)

Text S1. Calculation of the absolute photoluminescence (PL) quantum yield.

The absolute PL QY aqueous solution of samples was measured with an Edinburgh FS5 fluorescence spectroscope. During quantum yield testing, the sample concentration was 0.8 mg/mL, the excitation wavelength was 358 nm, and the solvent in this experiment was ultrapure water. The QY of samples was determined by using the following equation [47]:

$$QY=\frac{\int Iemission}{\int Iblank - \int Isample}$$

where $I_{emission}$ is the PL emission of CQDs aqueous solution, I_{blank} is the spectrum of the light used for excitation in the presence of solvent only, I_{sample} is the spectrum of the light used to excite the CQDs aqueous solution. Excitation range: 348-371 nm, fluorescence range: 372-507 nm. They were collected by using the integrating sphere.

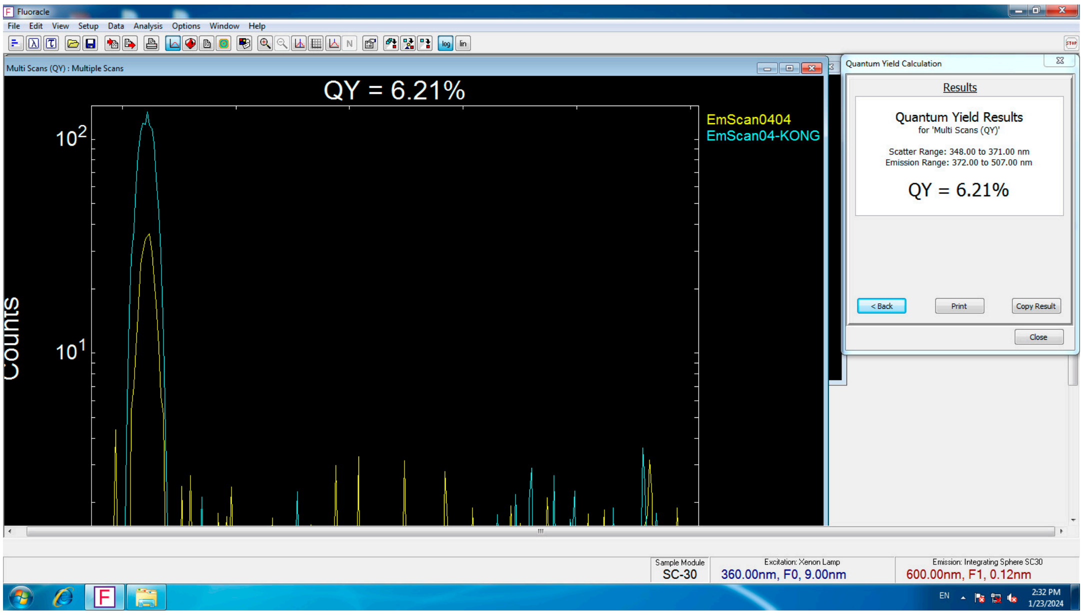

Figure S1. Calculation of absolute PL quantum yield of CQDs aqueous solution.
